# The Effects of the CCR6/CCL20 Biological Axis on the Invasion and Metastasis of Hepatocellular Carcinoma

**DOI:** 10.3390/ijms15046441

**Published:** 2014-04-16

**Authors:** Dongshu Du, Yeliu Liu, Haixin Qian, Bo Zhang, Xiaojun Tang, Ti Zhang, Weidong Liu

**Affiliations:** 1 College of Life Science, Shanghai University, 99 Shangda Road, Shanghai 200444, China; E-Mail: sdhzdds@163.com; 2 Department of General Surgery, Huai’an First People’s Hospital, Nanjing Medical University, 6 Beijing Road West, Huai’an 223300, Jiangsu, China; E-Mails: tangxiaojuntxj@126.com (X.T.); zhangti1982@126.com (T.Z.); liuwendong@126.com (W.L.); 3 Department of General Surgery, The First Affiliated Hospital of Soochow University, No. 188, Shizi Street, Suzhou 215006, Jiangsu, China; E-Mail: zxdeng@163.com; 4 Department of General Surgery, Zhangjiagang Hospital Affiliated to Soochow University, Zhangjiagang 215600, Jiangsu, China; E-Mail: doczhangbo@163.com

**Keywords:** CCR6, CCL20, hepatocellular carcinoma, metastasis

## Abstract

Chemokines and their receptors have recently been shown to play major roles in cancer metastasis. Chemokine receptor 6 (CCR6) and its ligand, CCL20, were highly expressed in a variety of human cancers. In our present study, we aimed to clarify whether CCR6/CCL20 was correlated with the migration of hepatocellular carcinoma (HCC). RT-PCR and Western blot results showed that CCR6 was overexpressed in different invasive potential HCC cell lines (*p <* 0.05), while the expression of CCL20 had no obvious difference (*p >* 0.05). CCR6 was suppressed by siRNA in HCCLM6, and then the biological behaviors of HCCLM6 cells were observed. The results showed that the CCR6/CCL20 biological axis increased the capacity of proliferation and adhesion, as well as the chemotactic migration and the level of cytokines related to degraded extracellular matrix. In conclusion, these findings indicate that CCR6 indeed participates in regulating the migration and invasion of HCC, and it might become a prognostic factor of HCC.

## 1. Introduction

Hepatocellular carcinoma (HCC) ranks among the most common malignancies worldwide with a poor prognosis. Although there have been significant developments in diagnosis, surgical treatment and adjuvant therapies for HCC, metastasis is the dominating cause of death in the majority of patients, the overall five-year survival rate of which is limited to 5%–6% [1,2]. Surgery is the main treatment for HCC; however, the recurrence rate after curative treatment is still disappointingly high. Given the grim outlook of HCC, novel therapeutic targets of genes and new modalities of effective chemoprevention and treatment are highly awaited.

Chemokines are a family of low molecular weight (8–10 kDa) pro-inflammatory cytokines, which bind to chemokine receptors and sustain the migration of neutrophils, lymphocytes, monocytes, DC cells and so on. Recent studies [3,4] suggested that chemokine receptors expressed highly on cancer cells might play a role in tumor progression and metastasis. The actin polymerization and pseudopodia formation within the cells may participate in this process, and the combination of chemokines and their receptors plays a very important role in determining the metastatic destination of tumor cells. Support for this theory comes from the overexpression of chemokine receptors CCR_7_ and CXCR_4_ in breast cancer cells, and the expression levels of their ligands (SDF-1 and 6) are much higher in the usual metastatic destination (lymph nodes, lung and liver) than the rare one (small intestine, skin, brain tissue and skeletal muscle) [5]. Lu [6] found that the expression level of CCR2 is much higher in cell stains that have stronger invasiveness, by the detection of CCR2 mRNA and protein expression levels in prostate cancer cells. As for providing a new research direction in anti-tumor therapy, organ selective transfer has become a hot spot in the field of tumor metastasis.

Chemokine receptor 6 (CCR6) is a member of the CC chemokine receptor family, whose main expression is observed in the spleen, lymph nodes, thymus and fetal liver tissue [7]. CCL20, a ligand of CCR6, is also known as a liver and activation-regulated chemokine and as MIP-3α. Immunohistochemical analysis of 64 colorectal cancer cases [8] found that the expression of CCR6 is closely associated with the hepatic metastasis of colorectal cancer, prompting that CCR6 and its ligands are involved in this process. Moreover, it is in thymus and ovarian cancer [9]. It is speculated that CCR6-mediated chemotaxis may be a common mechanism of malignant tumor metastasis, suggesting that CCR6 signaling pathway suppression may prevent the risk of liver metastasis.

Hepatic metastase, a highly organized and non-random organ-specific process, includes several sequential steps. It may develop not only as a result of intrahepatic metastasis, but also hematogenous metastasis, within which pulmonary distant metastasis is common. Speculation is reasonable that CCR6 and its ligand CCL20 may be determinant in intrahepatic metastasis [10] and distant migration of lungs based on the constitutive expression of CCL20 and CCR6 in the liver, lungs and other organs.

Based on many previous reports [11–13], we suppose CCR6 may play major roles in hepatocellular carcinoma metastasis and participate in regulating the migration and invasion of HCC, and CCR6 expression would have some correlation with metastasis of HCC. In the present study, we examined the relationship of CCR6/CCL20 and the biological behavior of HCC, which would help us understand the mechanism of the invasion and metastasis of HCC.

## 2. Results and Discussion

### 2.1. CCR6/CCL20 Expression in Cell Lines

**Figure 1.**
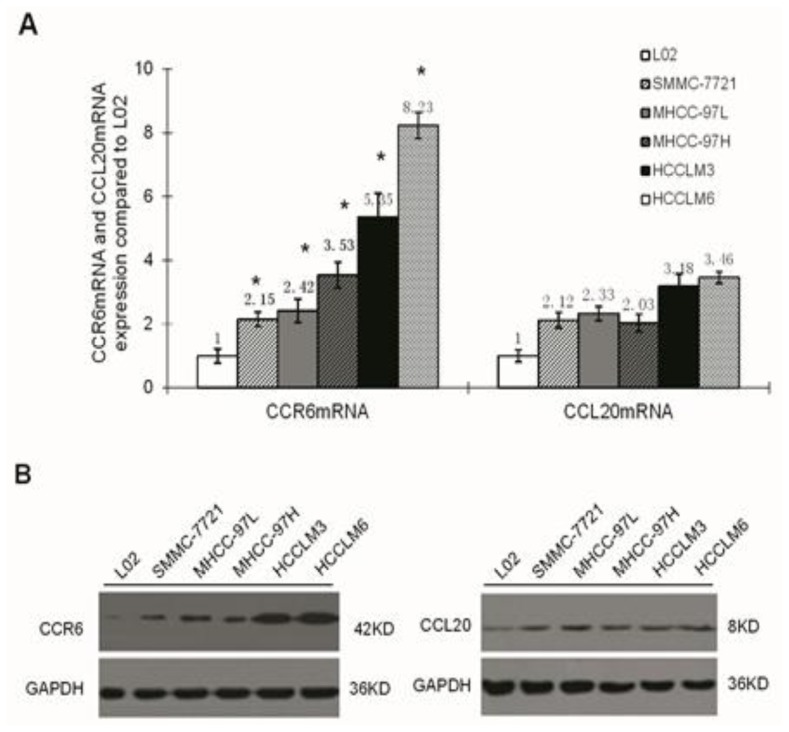
The mRNA (**A**) and protein (**B**) expression of CCR6 and CCL20 mRNA in five kinds of hepatocellular carcinoma cell lines (L-02, SMMC-7721, MHCC-97L, MHCC-97H, HCCLM3 and HCCLM6); * *p <* 0.05.

The expression of CCR6/CCL20 mRNA and protein in liver/liver cancer cell lines was detected by real-time PCR and Western blot, respectively. CCR6 expression was different in the five cell lines (SMMC-7721, MHCC-97L, MHCC-97H, HCCLM3 and HCCLM6) (*p <* 0.05) (Figure 1A), and it is the highest in the HCCLM6 cell line. However, the expression levels of CCL20 in these five kinds of cell lines have no obvious difference (*p >* 0.05) (Figure 1B).

### 2.2. Quantity of Plasmid DNA Affects Transient Transfection Rate

**Figure 2.**
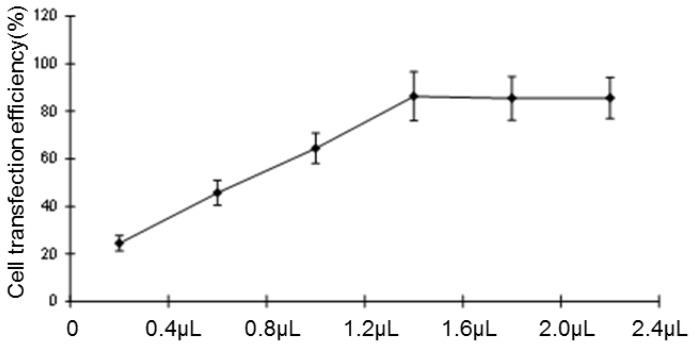
The transient transfection rate of the HCCLM6 cell line.

The transfection rate achieved 85% after transient transfection for 48 h. As shown (Figure 2), the plasmid DNA quantity of the highest transfection rate was 1.6 μL, above which the transfection rate has no corresponding increase. This revealed that 1.6 μL is the most suitable quantity for transfection.

### 2.3. Screen Effective siRNA Sequence

**Figure 3.**
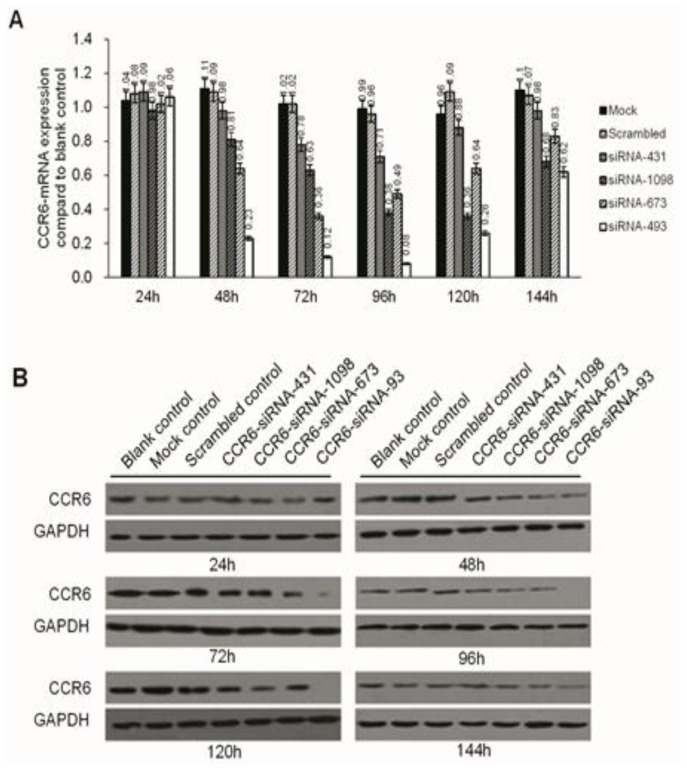
CCR6 mRNA and protein level in HCCLM6 cells were detected by real-time PCR after transfection for 24, 48, 72, 96, 120 and 144 h. (**A**) All the interference groups have various degrees of depressive effects after transient transfection for 48 h, and the depressive effect reached a peak after 72–96 h. The level of CCR6 mRNA in the CCR6-siRNA-493 group knockdown was most (*p <* 0.05). The mock group is used as reference; (**B**) The level of CCR6 protein in the CCR6-siRNA-493 group is depressed most (*p <* 0.05) and reached the peak after 96–120 h.

We first evaluated the capacity of anti-CCR6 siRNAs directed against CCR6 mRNA to inhibit CCR6 expression in HCCLM6 cell lines. CCR6-siRNA-493 inhibited the expression of CCR6 in HCCLM6 cell lines in gene and protein levels significantly, compared to that of the mock control group, the scrambled control group and the CCR6-siRNA-431 (-673, 1098, -1098) groups, as shown in Figure 3.

### 2.4. Knockdown of CCR6 Inhibits Both the Proliferation and Adhesion Ability of HCCLM6 Cells

**Figure 4.**
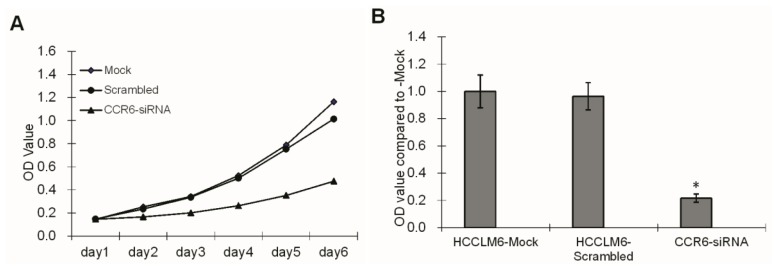
Proliferation and adhesion ability of HCCLM6 cells. (**A**) The cell proliferation rate of the CCR6-siRNA transfection group was obviously lower than that of HCCLM6-mock control group and the HCCLM6-scrambled control group from the second day after inoculation (* *p <* 0.05); (**B**) Compared to the HCCLM6-Mock control group, the adhesion ability of the HCCLM6 cells in the CCR6-siRNA group decreased obviously (* *p <* 0.05). OD, optical density.

The proliferation and adhesive ability are the most important functions of HCCLM6 cells, both *in vivo* and on the implanted device surface, which can be measured via 3-(4,5-dimethylthiazol-2-yl)-2,5-diphenyl tetrazolium bromide (MTT) (Figure 4A) and cell adhesion experiments (Figure 4B). We found that CCR6 gene silence inhibits the proliferation and adhesion ability of HCCLM6 cells.

### 2.5. CCR6-siRNA Reduced Migration and Invasive Ability of HCCLM6 Cells

**Figure 5.**
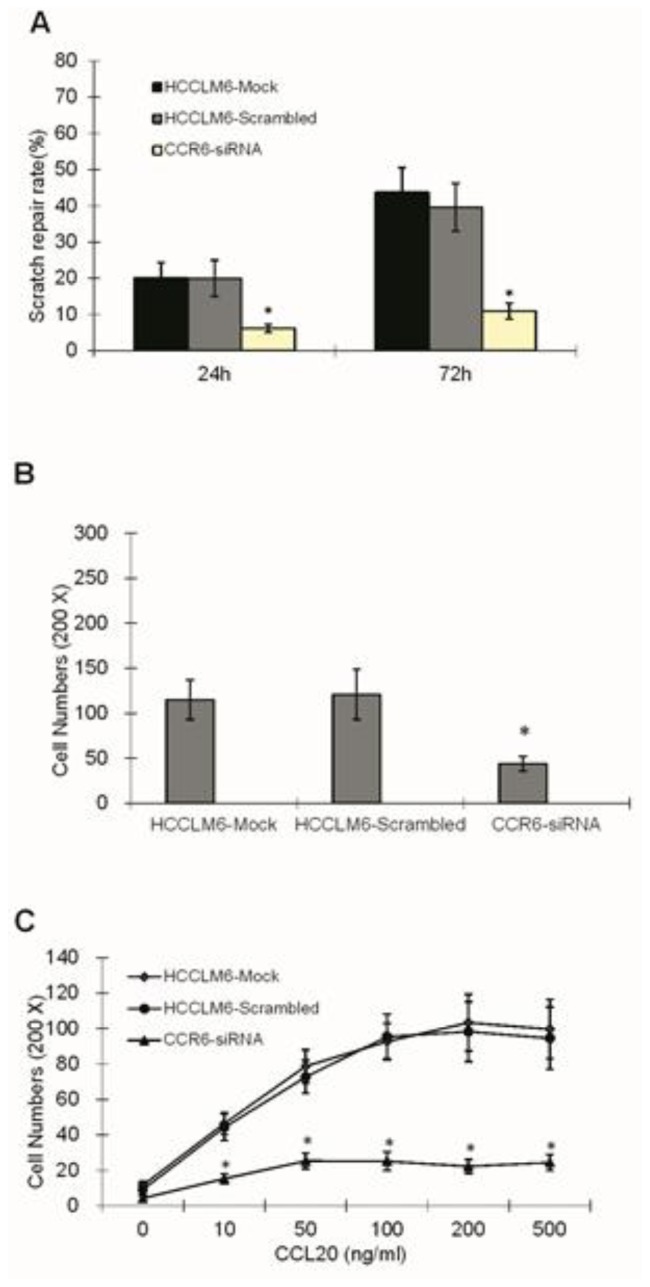
The scratch repair rates (**A**); invasive ability (**B**) and chemotaxis of cells (**C**) in different groups. * *p <* 0.05 compared with the control groups.

The migration rate was measured by the wound healing assay. Twenty four hours after scratching, the rate of the scratch wound repair in the HCCLM6-mock, HCCLM6-scrambled and HCCLM6-siRNA groups were 21.14% ± 4.21%, 20.00% ± 5.03% and 6.16% ± 1.12% respectively. Additionally, 72 h after scratching, the rate of the scratch wound repair rose to 43.75% ± 6.83%, 39.57% ± 6.63% and 10.96% ± 2.23% (Figure 5A). This result demonstrated that the inhibition of CCR6 expression decreased the migration ability of HCCLM6 cells. There is a good correlation between the migration ability *in vitro* and the invasive ability *in vivo*, which is represented by the power to traverse cell membranes. The invasive ability of HCCLM6 cells was measured by the matrigel invasion assay; the cells that could transverse the artificial basement membrane were quantified by using an inverted microscope. Compared with the control groups, the value in the CCR6-siRNA group was much lower (*p <* 0.05) (Figure 5B). As Figure 5C shows, chemokine CCL20 induced HCCLM6 cells across the filter membrane in a concentration-dependent manner in the HCCLM6-mock group and the HCCLM6-scrambled group, but not in the CCR6-siRNA group.

### 2.6. The Effects of CCR6-siRNA on The Expression of Cytokines, Proliferation Related-Protein and Metastasis Related-Protein

**Figure 6.**
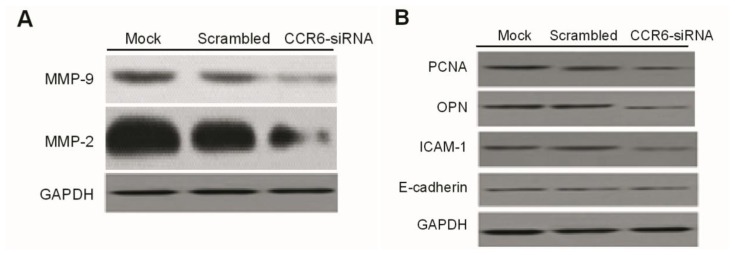
The release of MMP-2, MMP-9 (**A**) and the expression of PCNA, ICAM-1 and OPN (**B**) in the HCCLM6-mock group, the HCCLM6-scrambled group and the CCR6-siRNA group.

The expression of MMP-1 and MMP-9 was detected by gelatin zymography. Negative stripes of activated MMP-2 and MMP-9 enzyme were visible in the HCCLM6-mock group and the HCCLM6-scrambled group. However, the expression of MMP-2 and MMP-9 was reduced in the CCR6-siRNA group (Figure 6A).

The expression of proliferation, invasion and metastasis-related proteins, such as PCNA, ICAM-1 and OPN, was detected by Western blot. Compared to the HCCLM6-mock group and the HCCLM6-scrambled group, the expression of PCNA, ICAM-1 and OPN significantly decreased in the CCR6-siRNA group. However, the expression of E-cadherin was not changed among all the groups (Figure 6B).

### 2.7. Discussion

Cancer metastasis is a dynamic process that involves several sequential steps and is a result of the coaction between the cell and extracellular matrix. It has been proposed that key factors regulating the metastasis of tumor cells are the abilities of adhesion, metastaticity and degradation. Although several molecules [14,15] are reported to be involved in cancer metastasis, chemokines and their receptors have recently been shown to have a major role. The interaction between them cause cell membrane folding and pseudopodia formation, so as to induce cell migration, falling from the primary site, penetrating the vascular basement membrane and eventually entering the circulation and transferring to the target organs [5].

In the present study, we investigated the role of CCR6 in the metastasis of HCC. As in a recent report [16], the expression of CCR6/CCL20, which is associated with the malignant biological behavior of HCC, increases significantly in cancer tissue. Support for this theory comes from the greater tendency of the occurrence of vascular invasion, intrahepatic metastasis, pulmonary metastasis and other malignant activities in CCR6-positive HCC patients [10,16], after adjustment for the clinical influence factors of gender, age, tumor size and degree of differentiation. That is to say, abnormally high expression of CCR6/CCL20 may be an important factor participating in the occurrence and development of liver cancer. In the present study, we quantified the expression of CCR6/CCL20 in cell lines with different metastatic potential by RT-PCR and Western blot. The results indicated that the expression quantity of CCR6 increases with metastasis potential. As for CCL20, there was no obvious differences. The investigation of the relationship between CCR6/CCL20 expression and liver cancer prognosis showed that CCR6 was closely associated with the overall survival rate of liver cancer, which had no significant correlation with CCL20. Thus, it may be possible to inhibit the metastasis of HCC by blocking CCR6 with an antibody, antagonist or siRNA.

The identification of overexpression molecules on cancer cells may help us to explore the targets whose downregulation can elicit significant benefits for cancer treatment. The RNA interference mechanism, particularly the chemically synthesized small-interfering RNAs (siRNAs), which have the ability to mediate the degradation of any mRNA with a complementary nucleotide sequence in a specific manner, can efficiently downregulate the given genes. To clarify the influence of CCR6 on the biological behavior of HCCLM6 cells, a pGPH1/GFP/Neo-CCR6-siRNA plasmid was established. For Neo genes endowed with the successfully transfected cell G418 resistance, G418 was used to screen for stable transfected cell strains in the present study. Among the alternative options, CCR6-siRNA-493 inhibited the expression of CCR6 in genes and protein levels most significantly, which was verified by RT-PCR and Western blot.

Through blocking the expression of CCR6 in HCCLM6, the proliferation, invasion and metastasis abilities of liver cancer cells decreased, as did the migration ability. Meanwhile, suppression of CCR6 expression significantly reduced tumor cell chemotaxis of its specific ligand, CCL20, the impact of which is dose dependent in untreated HCCLM6 cells. The mechanism may be that the specific binding of CCR6 and CCL20 activates the downstream signaling pathways, which inhibit the expression of genes and proteins related to invasion and metastasis. The results confirmed that CCR6, which is expressed in cell membranes and cytoplasm, is a potential target to inhibit the invasion and metastasis of HCC. It also prompted the CCR6 signaling pathway as preventing the risk of liver metastasis.

In conclusion, we found that CCR6 is an important factor in the metastasis of HCC and a possible prognostic factor in early onset. Meanwhile, further studies may be needed to provide multiple therapeutic strategies to inhibit the metastasis of HCC.

## 3. Experimental Section

### 3.1. Cell Lines and Cell Culture

Normal liver cell line L02 and different invasive potential HCC cell lines, SMMC-7721, MHCC-97L, MHCC-97H, HCCLM3 and HCCLM6, were used. All kinds of cells were maintained in DMEM with high sugar (Gibco, Darmstadt, Germany) containing 10% fetal bovine serum (Gibco), at 37 °C in a 5% CO_2_ humidified atmosphere.

### 3.2. RNA Oligos and Transfection

Negative miRNA control (miR-NC), HCCLM6-mock (liposomes only), HCCLM6-scrambled control (irrelevant sequence with the same base arrangement as siRNA) and siRNA of CCR6 (pGPH1/GFP/Neo-CCR6-siRNA-431, -1098, -673, -493) were purchased from Gene Pharma (Shanghai, China). About 12–24 h before transfection, the cells were seeded in 24-well culture plates (0.5 × 10^5^/well). At the time of transfection, cells were 80% confluent. The complete medium was replaced by serum-free medium and incubated at 37 °C with 100 μL of siRNA complex solutions at the indicated concentration. After 24 h, the medium was removed, and the cells were cultured for 24 h in complete culture medium (with 10% FBS) without any transfection reagents. The transfection efficiency was evaluated by the number of cells expressing green fluorescent protein with a fluorescence microscope. Due to the carrying of the Neo gene in the pGPH1/GFP/Neo plasmid, which endowed G418 resistance to the successful transfected cells, G418 (Gibco) was used to screen for stable, transfected strains. The expression of CCR6 in the level of gene and protein were then screened for the most effective siRNA sequence.

### 3.3. Real-Time PCR

RNA was extracted using the RNeasy Mini kit and synthesized for cDNA. Quantitative real-time PCR was conducted in an SYBR Green PCR Kit using the ABI Prism 7500 Real-time PCR System, and each sample was run in triplicate.

### 3.4. Western Blotting

Western blotting was done as previously described. Briefly, at the end of the designated experiments, cell lysates were separated with 10% SDS-PAGE and clear protein extracts were obtained by centrifugation for 30 min at 4 °C. Twenty to forty milligrams of protein mixed with loading buffer were loaded per lane, separated by 12% SDS-polyacrylamide gel electrophoresis (SDS-PAGE). Proteins were transferred to PVDF membrane filters. After each membrane was blocked with 5% milk for 1 h at room temperature (RT), it was probed with specific monoclonal antibodies (Abcam, Cambridge, MA, USA) overnight at 4 °C. After washing with phosphate-buffered saline (PBS), the membranes were incubated with corresponding secondary antibodies (Abcam) TBST-5% nonfat milk for 1 h at RT. The immuno-reactive bands were visualized by enhanced chemiluminescence reagent, and GAPDH served as the loading control.

### 3.5. MTT Assay

HCCLM6 cell proliferation was tested using a 3-(4,5-dimethylthiazol-2-yl)-2,5-diphenyl tetrazolium bromide (MTT) assay. For the assay, three groups of cells, HCCLM6-mock, HCCLM6-scrambled and CCR6-siRNA, were freshly isolated and plated in 96-well flat bottom cell culture plates at a concentration of 1 × 10^5^ cells/well containing 100 μL of DMEM (supplemented with 10% FBS) culture medium. At 2, 3, 4, 5 and 6 d of culture, 20 μL of MTT solution (Sigma, St. Louis, MO, USA) in a concentration of 5 mg/mL was added per well. After 4 h of incubation, the culture medium was dissolved with 150 μL DMSO (Sigma, St. Louis, MO, USA). Plates were kept on an orbital shaker for 10 min and the optical density (OD) was read on a multiwell scanning spectrophotometer at 570 nm.

### 3.6. Adhesion Assay

Logarithmic growth of cells in three groups, HCCLM6-mock, HCCLM6-scrambled and CCR6-siRNA, were cultured in DMEM (free of serum) for 24 h and resuspended by serum-containing medium. Suspensions of cells were seeded in 96-well plates at a density of 4 × 10^4^/well, which were coated with Matrigel (Invitrogen, Carlsbad, CA, USA) and incubated in 10% FCS for 1h. After being cultured at 37 °C in 5% CO_2_ humidified atmosphere for another 1 h and being washed twice with PBS, each well of the 96-well plates was added to 20 μL fresh MTT solution in a concentration of 5 mg/mL. Then, the medium was replaced by 150 μL DMSO after 2 h. Plates were shaken in a dark place for 10 min, and the optical density (OD) was recorded at 570 nm.

### 3.7. Fibronectin Adhesion Assay

In this assay, 96-well plates were coated with 100 μL fibronectin (Sigma, St. Louis, MO, USA) overnight at 4 °C. As a positive control, holes were coated with 1% BSA, and for negative control, holes were coated with 100 μL/mL polylysine. The optimal concentrations of BSA and polylysine were chosen empirically by titration. Then, the plates were blocked for 30 min at 37 °C with 200 μL of 1% BSA and washed with PBS. After being subsequently immersed in cell culture media with serum-free high glucose DMEM, the wells were seeded by the suspension of HCCLM6 cells with 5.0 × 10^4^ cells/well and then maintained at 37 °C in a humidified atmosphere containing 5% CO_2_ for 30 min. After removal of the culture medium, the samples were fixed with ethanol for 10 min and washed with PBS. Crystal violet hydrate solution was transferred to the 96-well plates for 25 min. After being rinsed with water, each well was added to 200 μL of solubilization solution containing 0.5% TrtionX-100 and shaken overnight. The absorbance in 570 nm of the samples were evaluated by an enzyme mark instrument.

### 3.8. Wound Healing Assay

Suspension of cells in three groups (HCCLM6-mock, HCCLM6-scrambled and CCR6-siRNA) were plated on 6-well plates with a concentration of 5 × 10^5^/mL, the plates of which had been smeared by Matrigel glue and incubated in 10% FBS for 1 h. Until confluence with a monolayer of cells, each well was infused with fresh DMEM (free of FBS) for 24 h and scratched in a reproducible way using a sterile 200 μL pipette tip. The same wound area was photographed at once under a Zeiss Axioskop microscope and was also done after being cultured in DMEM (supplemented with 10% FBS) for 24 and 72 h. The distance between the edges of the scratch defect were measured and averaged from four separate experiments.

### 3.9. Matrigel Invasion Assay

A manmade Matrigel invasion chamber (Corning Corporation, Midland, MI, USA) was used according to the manufacturer’s instruction. The upper portion of the transwell chamber was coated with 100 μL Matrigel with 2.0 mg/mL (dissolved with serum-free DMEM) and incubated at 37 °C for 3 h. Then, 1 × 10^6^/mL cells (previously starved in serum-free DMEM for 24 h) in the groups of HCCLM6-mock, HCCLM6-scrambled and CCR6-siRNA were trypsinized, washed, resuspended and added to the upper chamber in serum-free DMEM, and migration at 37 °C towards 15% FBS containing growth media was determined after 24 h. Cells invading through the Matrigel and migrating to the bottom chamber were fixed with 4% paraformaldehyde, stained with 1% hematoxylin-eosin staining (HE), photographed and counted.

### 3.10. Chemotaxis Assay

Cells resuspended by DMEM (free of serum) to a concentration of 1 × 10^6^/mL were seeded in the upper chamber. The lower chamber was covered with chemoattractant CCL20 (R&D Systems, Minneapolis, MN, USA) diluted by serum-free DMEM, whose concentration gradient was 0, 10, 50, 100, 200 and 500 ng/mL. After incubation for 24 h at 37 °C with 5% CO_2_, cells on the lower surface of the membrane were stained and counted under a light microscope (×200). Assays were performed in triplicate.

### 3.11. Gelatin Zymography

To evaluate the nature of the enzymes responsible for the observed gelatinolytic activity, 2 × 10^5^/mL cells in the groups of HCCLM6-mock, HCCLM6-scrambled and CCR6-siRNA were vaccinated in 6-well plates. After being cultivated in serum-free DMEM for 24 h, proteins were then extracted and the concentration adjusted to 2.0 mg/mL. SDS-PAGE substrate zymography was carried out with separating gels containing 1 mg/mL of gelatin. Ten microliters of extract were mixed with an equal volume of 2× loading buffer, and then, the mixture was applied to each well of the gel. After electrophoresis was complete, which was then run at 20 mA at 4 °C, the gel was washed twice in washing buffer for 30 min and incubated in developing buffer for 18 h at 37 °C. Gels were stained with 0.25% Coomassie brilliant blue solution and then destained with 30% methanol and 10% acetic acid. Gelatinolytic activities were detected as unstained bands against the background of Coomassie blue-stained gelatin.

### 3.12. Statistical Analysis

By using the software SPSS16.0 (Chicago, IL, USA), data were presented as the mean ± SEM, and statistical differences were evaluated by one-way analysis of variance, comparison of positive difference with chi-square test (continuous correction). Image analysis of graphics was measured by Image Pro-Plus 6.0 software. For all analyses, we considered *p <* 0.05 to be statistically significant.

## 4. Conclusions

CCR6 regulates the migration and invasion of HCC. Even more, CCR6 might be able to become one of the prognostic factors for HCC.

## References

[1] Hu B., Tian X., Sun J., Meng X. (2013). Evaluation of individual and combined applications of serum biomarkers for diagnosis of hepatocellular carcinoma: A meta-analysis. Int. J. Mol. Sci.

[2] Han M.S., Moon K.S., Lee K.H., Cho S.B., Lim S.H., Jang W.Y., Jung T.Y., Kim I.Y., Jung S. (2013). Brain metastasis from hepatocellular carcinoma: The role of surgery as a prognostic factor. BMC Cancer.

[3] Wyler L., Napoli C.U., Ingold B., Sulser T., Heikenwälder M., Schraml P., Moch H. (2013). Brain metastasis in renal cancer patients: Metastatic pattern, tumour-associated macrophages and chemokine/chemoreceptor expression. Br. J. Cancer.

[4] Pevida M., Lastra A., Meana A., Hidalgo A., Baamonde A., Menéndez L. (2014). The chemokine CCL5 induces CCR1-mediated hyperalgesia in mice inoculated with NCTC 2472 tumoral cells. Neuroscience.

[5] Müller A., Homey B., Soto H. (2001). Involvement of chemokine receptors in breast cancer metastasis. Nature.

[6] Lu Y., Cai Z., Xiao G. (2007). CCR2 expression correlates with prostate cancer progression. J. Cell. Biochem.

[7] Schutyser E., Struyf S., Van D.J. (2003). The CC chemokine CCL20 and its receptor CCR6. Cytokine Growth Factor Rev.

[8] Ghadjar P., Coupland SE., Na I.K. (2006). Chemokine receptor CCR6 expression level and liver metastases in colorectal cancer. J. Clin. Oncol.

[9] Dellacasagrande J., Schreurs O.J., Hofgaard P.O. (2003). Liver metastasis of cancer facilitated by chemokine receptor CCR6. Scand. J. Immunol.

[10] Uchida H., Iwashita Y., Sasaki A. (2006). Chemokine receptor CCR6 as a prognostic factor after hepatic resection for hepatocellular carcinoma. J. Gastroenterol. Hepatol.

[11] Fuji H., Itoh Y., Yamaguchi K. (2004). Chemokine CCL20 enhance the growth of HuH7 cells via phosphorylation of 044/p42 MAPK *in vitro*. Biochem. Biophys. Res. Commun.

[12] Murphy P.M. (2001). Chemokines and the molecular basis of cancer metastasis. N. Engl. J. Med.

[13] Wang J.M., Deng X., Gong W. (1998). Chemokines and their role in tumor growth and metastasis. J. Immunol. Methods.

[14] Thomassen M., Tan Q., Burton M., Kruse T.A. (2013). Gene expression meta-analysis identifies cytokine pathways and 5q aberrations involved inMetastasis of ERBB2 amplified and basal breast cancer. Cancer Inform.

[15] Kwon M., Lee S.J., Wang Y., Rybak Y., Luna A., Reddy S., Adem A., Beaty B.T., Condeelis J.S., Libutti S.K. (2013). Filamin A interacting protein 1-like inhibits WNT signaling and MMP expression to suppress cancercell invasion and metastasis. Int. J. Cancer.

[16] Hu D., Du C., Xue W., Dou F., Yao Y., Gu J. (2013). The expression of chemokine receptors CCR6, CXCR2 and CXCR4 is not organ-specific for distant metastasis in colorectal cancer: A comparative study. Histopathology.

